# Revisiting NTRKs as an emerging oncogene in hematological malignancies

**DOI:** 10.1038/s41375-019-0576-8

**Published:** 2019-09-24

**Authors:** Sunil K. Joshi, Monika A. Davare, Brian J. Druker, Cristina E. Tognon

**Affiliations:** 1 Knight Cancer Institute, Oregon Health & Science University, Portland, OR, United States; 2 Department of Physiology & Pharmacology, School of Medicine, Oregon Health & Science University, Portland, OR, United States; 3 Division of Hematology & Medical Oncology, Department of Medicine, Oregon Health & Science University, Portland, OR, United States; 4 Papé Pediatric Research Institute, Oregon Health & Science University, Portland, OR, United States; 5 Division of Pediatric Hematology & Oncology, Department of Pediatrics, Oregon Health & Science University, Portland, OR, United States; 6 Howard Hughes Medical Institute, Oregon Health & Science University, Portland, OR, United States

## Abstract

NTRK fusions are dominant oncogenic drivers found in rare solid tumors. These fusions have also been identified in more common cancers, such as lung and colorectal carcinomas, albeit at low frequencies. Patients harboring these fusions demonstrate significant clinical response to inhibitors such as entrectinib and larotrectinib. Although current trials have focused entirely on solid tumors, there is evidence supporting the use of these drugs for patients with leukemia. To assess the broader applicability for Trk inhibitors in hematological malignancies, this review describes the current state of knowledge about alterations in the NTRK family in these disorders. We present these findings in relation to the discovery and therapeutic targeting of BCR–ABL1 in chronic myeloid leukemia. The advent of deep sequencing technologies has shown that NTRK fusions and somatic mutations are present in a variety of hematologic malignancies. Efficacy of Trk inhibitors has been demonstrated in NTRK-fusion positive human leukemia cell lines and patient-derived xenograft studies, highlighting the potential clinical utility of these inhibitors for a subset of leukemia patients.

## Introduction

Advances in technologies from chromosomal banding to massively parallel sequencing have enabled the identification of oncogenic mutations, and enhanced our understanding of the biology contributing to malignant phenotypes. A seminal example is the identification of the BCR–ABL1 fusion protein in chronic myeloid leukemia (CML) [1–4]. Studies of BCR–ABL1 have not only shaped our understanding of the tumorigenic process but also provided insight into how cancer can be treated. The discovery and success of imatinib, the first FDA-approved tyrosine kinase inhibitor against BCR–ABL1, has revolutionized how we approach the treatment of cancer [5, 6]. Importantly, this paved the way for precision oncology, wherein development of selective, molecularly-guided therapeutic modalities have shown significant improvements in patient outcomes, as compared with nonselective chemotherapeutics. This principle has also been shown to be effective in the treatment of several solid and liquid tumors [7–9], underscoring the broad value of using drugs that precisely target cancer driving lesions.

Recently, the FDA granted accelerated approval to larotrectinib (also commonly referred to as LOXO-101 or Vitrakvi^™^), the first selective neurotrophic tyrosine receptor kinase (NTRK) inhibitor for patients of all ages with advanced solid tumors harboring NTRK gene fusions, regardless of tumor histology [10, 11]. The efficacy and safety profile of larotrectinib were confirmed in three independent trials with patients ranging from all ages (the youngest being a 1-month-old) [11–13]. Interestingly, NTRK gene fusions occur at higher frequency (up to 90%) in patients with rare cancers, such as infantile fibrosarcoma, secretory breast carcinoma, mammary analogue secretory carcinoma, and cellular or mixed congenital mesoblastic nephroma, but are less prevalent in common adult tumors [14]. With an overall response rate of >75%, a median duration of response not reached following 18 months, and minimal adverse effects, larotrectinib’s efficacy parallels that of imatinib, and marks another milestone for the field of precision oncology [12]. Even more recently, entrectinib, a pan-Trk, ROS1, and ALK inhibitor also received accelerated FDA approval [15]. Similar to imatinib, the approval of larotrectinib and entrectinib remind us of the importance of understanding the biological target as a stringently vetted response biomarker, and the need to continue screening for other actionable targets by harnessing the rapidly amassing ‘omics data.

Moreover, with the approval of larotrectinib and entrectinib, the Trk family of cell surface tyrosine kinase receptors have drawn considerable attention. NTRK1, 2, and 3 genes encode TrkA, TrkB, and TrkC receptors, respectively. These receptors signal through JAK/STAT, PI3K/AKT, and MEK/ERK to promote proliferation, differentiation, and survival [16, 17]. Although much of the literature has focused on the importance of these receptors in central and peripheral nervous system development and function [18], alterations in the NTRK family have been described in colon [19], thyroid [20, 21], lung [22], glial [23], and breast [24] cancers. These alterations are found at relatively low frequencies (<1%) within each of these individual solid tumors but collectively, when considering all tissues, NTRK-driven cancers constitute a significant number of patients, making them an important therapeutic target [22, 25–28]. Notably, NTRK fusions are pathognomonic for several rare solid tumor malignancies [24, 26, 29–33]. A number of excellent reviews have summarized recent work on Trk signaling in solid tumors [14, 16, 34, 35]. Despite the emerging success of NTRK inhibition in solid tumors, the role of these receptors in hematologic malignancies remains under investigated. Therefore, this review provides a comprehensive overview of our current understanding of NTRK-mediated tumorigenesis in hematological malignancies and links recent successes in NTRK-targeted therapy to historical milestones achieved by the targeting of BCR–ABL1 in CML.

## NTRK receptor alterations and their role in cancer development

To date the BCR–ABL1 fusion remains the most prevalent mechanism of oncogenic ABL activation. In contrast activation of NTRK receptors can result from a wider range of molecular events such as chromosomal rearrangements, deletions/truncations, point mutations, and changes in mRNA and protein expression. Among these mechanisms, oncofusions involving NTRK receptors are the most common mechanism of activation.

### NTRK oncofusions

**Fig. 1:**
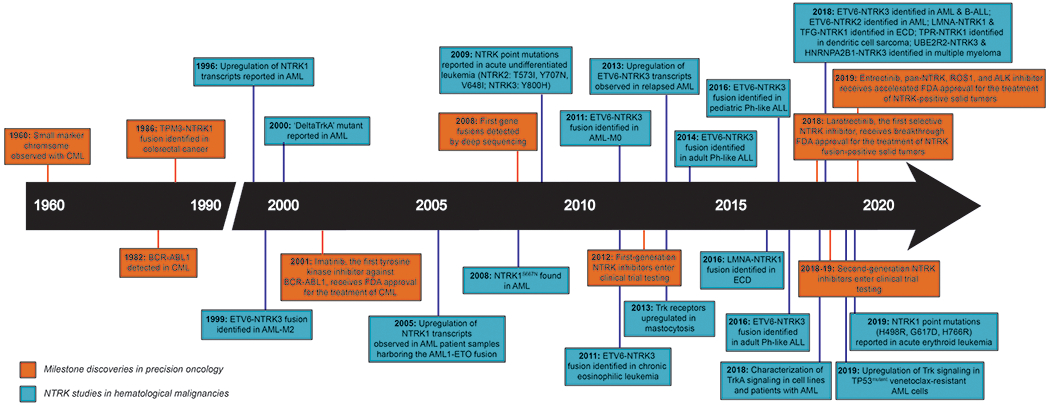
This timeline highlights the discovery of NTRK-related alterations in a variety of hematologic malignancies in relation to milestone studies that ushered an era of precision oncology. The growing number of NTRK alterations found in hematologic malignancies over the years suggests that these alterations have profound clinical implications that warrant further investigation

In 1986, shortly after the confirmation of BCR–ABL1 in CML [2], the first gene fusion involving an NTRK receptor was identified in a patient with colorectal cancer (Fig. 1). This oncogenic translocation, TPM3-TRK, resulted from the fusion of the tropomyosin 3 gene amino terminus with the transmembrane and kinase domains of NTRK1 [19], a finding that has since been confirmed [36]. Over the past few years, several NTRK fusions have been reported in solid tumors [34, 35, 37, 38].

Many of these Trk fusions involve the transcription factor, E26 transformation-specific variant 6 (ETV6, also known as TEL) located on chromosome 12p13. Specifically, ETV6–NTRK3 fusions (EN; t(12;15) (p13;q25)) have been previously characterized in the setting of secretory breast carcinoma [24, 39], congenital fibrosarcoma [40], thyroid carcinoma [41], and pontine gliomas [23]. Interestingly, the EN fusion is the first oncogenic fusion to be identified in cancers that are derived from all three cell lineages [35]. In the most common version of the EN fusion, the amino terminus of the ETV6 transcription factor, containing the helix-loop-helix (HLH) domain (exons 1–5; also commonly referred to as the sterile alpha motif or pointed domain), fuses with the kinase domain of the partnering protein resulting in constitutive kinase activity [42, 43]. The ETV6 HLH domain has been shown to be essential for mediating protein activity of the EN fusion. Its deletion results in the loss of dimer formation and ability to transform mutant cells [44]. Substituting the ETV6 HLH domain with an inducible FK506 binding protein dimerization domain does not inhibit catalytic activation of the fusion, but abrogates its transformative capacity. These data suggested that the ETV6 HLH domain provides specific signaling or polymerization capabilities required for full activation of the fusion protein [45–47].

**Fig. 2:**
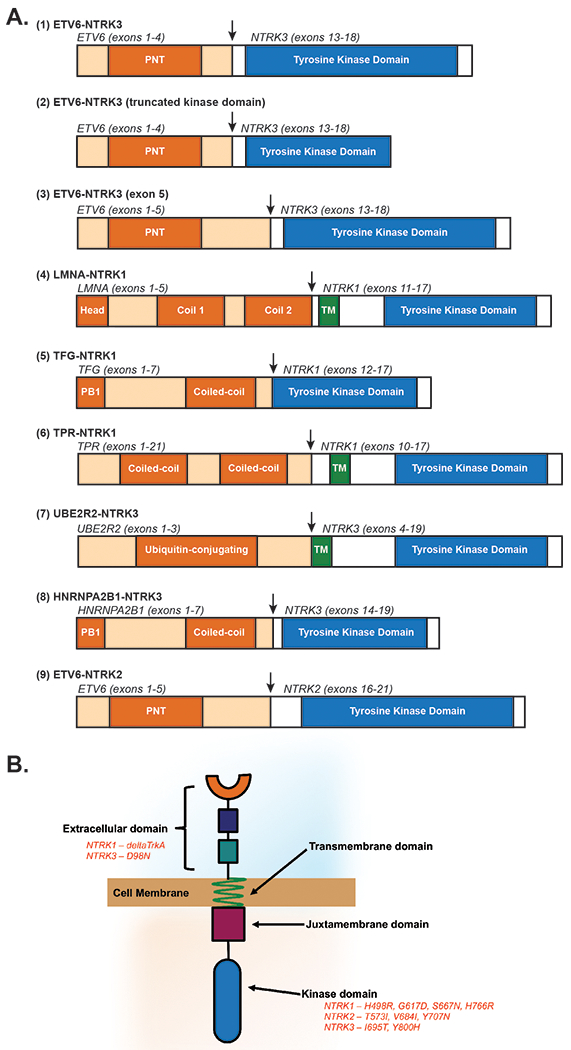
**a** Diagram of the unique Trk fusions identified in various hematological malignancies [52, 58, 66]. For each fusion, the carboxy-terminal kinase domain of the Trk protein is fused in-frame with the upstream amino-terminal binding partner. All relevant domains that contribute to the final chimeric fusion are shown. Vertical arrows indicate the breakpoint. PNT pointed domain, TM transmembrane domain. Fusions are numbered in the order they are discussed throughout the text. **b** Representative Trk receptor indicating location of known deletions [70] and point mutations [77–79]

Although other, non-NTRK ETV6-based fusions have been long reported to play a role in leukemogenesis [42, 48–51], an EN fusion was first reported in 1999 by Eguchi et al. [52, 53] in a 59-year-old female with AML-M2 using fluorescence in situ hybridization (FISH). They identified two variants of the fusion with FISH (Fig. 2a). In each case, exons 1–4 of the ETV6 HLH domain were fused in-frame with exons 13–18 of NTRK3 that encoded the protein-tyrosine kinase (PTK) domain. These fusions differed from the EN fusions described in solid tumors as only the first four exons of ETV6 were fused with the kinase domain of NTRK3. Moreover, one fusion included the entire PTK domain (encoding a 52 kD protein), while the other variant involved a truncated PTK domain (encoding a 38 kD protein). However, both of these chimeric proteins lack a 42-base-pair exon near the C-terminus of the NTRK3 protein that was reported thereafter in the EN oncofusion found in cases of congenital fibrosarcoma [52]. In addition to the 42-base-pair stretch, the fusion found in congenital fibrosarcoma contains ETV6 exons 1–5 versus 1–4 that are seen in EN fusions found in acute myeloid leukemia (AML). When these differing EN fusions (±ETV6 exon 5) were expressed in mice, only the variant without exon 5 produced leukemia, suggesting the importance of the ETV6 exon number in determining the disease phenotype [54].

Differences in structural makeup of these EN oncofusions may also affect downstream signaling. Subsequent mechanistic studies described that the protein coded by the 42-base-pair moiety decreases tyrosine kinase activity and impairs downstream signaling mediated by SHC and PLCγ [55, 56]. In other words, robust kinase activity and signaling through PLCγ was observed only in the setting of AML, resulting from the EN fusion. These data suggest a necessary role for activation of PLC-regulated pathways, including protein kinase C and/or calcium flux mediated signaling, in myeloid leukemogenesis.

Twelve years after the first report of EN in AML, the EN fusion was identified in an 82-year-old female who developed chronic eosinophilic leukemia following pancreatic carcinoma [57]. That same year, a different variant of the EN fusion transcript was identified in a 55-year-old male patient with AML-M0 [58]. Analysis of this variant suggested that the first five exons of ETV6 fused with the kinase domain of NTRK3 (Fig. 2a). This was in contrast to the oncofusion described by Eguchi et al. [52], where only the first four exons of ETV6 were fused to NTRK3.

In studying samples from patients with AML, Pemovska et al. (2013) saw an abundant EN transcript in a 37-year-old AML patient with a recurrent t(11;19)(q23;p13.1) translocation corresponding to the MLL–ELL fusion gene [59]. This patient had relapsed from three previous rounds of conventional chemotherapy. While a decrease in bone marrow blast count following treatment with dasatinib, sunitinib, and temsirolimus was observed, this response was short-lived. Resistance had developed due to the upregulation of EN and STRN–ALK fusions, which provided these tumor cells a bypass mechanism to evade death [59].

In 2014, Roberts et al. were the first to describe the EN fusion in a case of Philadelphia chromosome-like Acute Lymphoblastic Leukemia (Ph-like ALL) through genomic analyses [60]. They went on to characterize this fusion via a conditional knockin mouse model, and noted the development of aggressive lymphoid leukemia with complete penetrance and a median latency of 38 days, recapitulating human B-ALL [61]. Treatment with PLX7486 or larotrectinib for 12 weeks decreased tumor burden and splenic weight.

Another case of the EN fusion in adult Ph-like ALL has also been reported [62]. After performing cytogenetic, FISH, and whole-genome single-nucleotide polymorphism (SNP) analysis on 60 ALL cases, an EN fusion was identified in a 9-year-old boy with B-ALL [63]. SNP analysis revealed a breakpoint within intron 5 of ETV6 that fused with the breakpoint in the C-terminal of NTRK3, resulting in the formation of the EN oncofusion. Interestingly, their finding contradicts an earlier report from Alessandri et al. (2001) who found the presence of no EN transcripts in pediatric AML and ALL patients via qPCR analysis [64]. However, this discrepancy could suggest the need for a more integrative approach when screening for the presence of such fusions.

Apart from EN fusions, in recent years other NTRK fusions have been described and functionally evaluated in hematological malignancies. An LMNA–NTRK1 fusion was identified in a 27-year-old patient with Erdheim–Chester disease (ECD, Fig. 2a) [65]. This fusion resulted in the activation of MAPK and PI3K/AKT signaling. In addition to the LMNA–NTRK1 and EN fusions, other NTRK fusions in patients with ECD, AML, and multiple myeloma were recently reported [66].

Specifically, after performing targeted RNA sequencing on 7311 patients with hematologic malignancies, Taylor et al. discovered eight patients harboring NTRK oncofusions [66] (Fig. 2a). They identified a TFG–NTRK1 fusion in a 2-month-old patient with ECD, a TPR–NTRK1 fusion in a 20-year-old patient with interdigitating dendritic cell sarcoma, and UBE2R2–NTRK3 and HNRNPA2B1–NTRK3 fusions in 53- and 76-year-old patients with multiple myeloma. Between the two fusions discovered in multiple myeloma, only the UBE2R2–NTRK3 fusion was able to transform Ba/F3 cells. This could result from the differing number of NTRK3 exons present in the final oncofusion. Regardless of transformation capacity, all fusions identified were sensitive to Trk inhibition as assessed via mouse colony formation and cell viability assays.

In the same study, they also identified a previously unreported NTRK2 fusion (ETV6–NTRK2) in a 77-year-old male with AML and studied its transforming potential and downstream signaling in a cytokine-dependent murine hematopoietic cell line (Ba/F3 cells) and in a patient-derived xenograft model [66] (Fig. 2a). This fusion demonstrated robust PI3K–AKT signaling that was attenuated upon treatment with larotrectinib both in vitro and in vivo. Surprisingly, this patient also had a cooccurring KRAS Q61P mutation. The presence of cooccurring mutations with driver tyrosine kinase alterations has been rarely reported [67, 68]. Nonetheless, this scenario warrants that cooccurring mutations with NTRK alterations should be considered as they may pose a resistance liability and limit durability of the response.

A recent study investigating the contribution of NTRK fusions in neuroendocrine tumors reported six cases, originating from various anatomic sites that include the pancreas, uterus, and lung [68]. Interestingly, in three patients, the NTRK fragment was 5′ to its fusion partner. Although it is unusual for NTRK to be the upstream binding partner, this finding potentially implicates an unknown function of the 5′ region of NTRK that should be further studied. Thus far, no such NTRK fusions have been described in hematological malignancies.

While there was a lag in the identification of NTRK fusions in hematologic malignancies since the first case reported by Eguchi et al., [52] the advent of deep sequencing technologies [69] in 2008 accelerated the detection of such fusions (Fig. 1). In fact, due to such technological advances, we have learned that NTRK fusions (and that of orthologues, ROS1, and ALK), irrespective of tumor histology, are more common than previously speculated [37].

### Deletions/truncations

A deleted form of NTRK1 (named ‘deltaTrkA’) that lacks 75 amino acids in the extracellular domain (including the ligand-binding Ig2 domain) and four glycosylation sites adjacent to the transmembrane domain was reported in 2000 in an AML patient [70] (Fig. 2b). The in-frame deletion of these amino acids resulted in constitutive tyrosine kinase activity. This aberrant activity could partly be explained by the loss of glycosylation, which is known to inhibit kinase activity in TrkA [71] and by the loss of cysteine residues that affect the overall tertiary structure of the receptor [72]. Expression of deltaTrkA in cells resulted in activation of Ras/MAPK and PI3K/AKT signaling and transformation of fibroblasts, epithelial, and myeloid cells in vitro [70]. A later study reported that mice transplanted with myeloid 32D cells engineered to express deltaTrkA developed polyclonal AML that was mediated by PI3K and mTOR-raptor signaling [73].

These initial studies with deltaTrkA suggested that the extracellular domain contained a ‘regulatory switch’ that, when lost, resulted in constitutive kinase activation. Parallel studies in other tumor models also validated the regulatory function of the extracellular domain (i.e., Ig1 and 2 subdomains) in preventing spontaneous dimerization and kinase activation [74, 75]. Specifically, deletion of exons 6, 7, and 9 and of the functional IG-C1 and N-glycosylation subdomains of TrkA, resulting in TrkAIII splice variant, promoted neuroblastoma [76].

The importance of the extracellular domain in serving as a mediator of Trk activation could also explain why constitutive kinase activity is seen with Trk oncofusions. It may be the case that loss of the extracellular domain (that encompasses the autoinhibitory subdomains) in addition to the HLH domain [47] of the upstream binding partner enables constitutive kinase activation.

### Point mutations

After performing high-throughput resequencing of the kinase domain of 26 tyrosine kinase genes, a mutation in the kinase domain of NTRK1^S667N^ was reported in four patients with AML of 188 tested [77] (Fig. 2b). A year later, sequencing of leukemic cells in another study revealed four novel previously unreported point mutations in NTRK receptors of four patients (NTRK2: T573I, V684I, and Y707N; NTRK3: Y800H) [78]. Point mutations in the NTRK3 receptor—D98N and I695T—have also been reported in patients with B-cell lymphoma and myeloid leukemia, respectively (The Cancer Genome Atlas). Functional characterization of the aforementioned point mutations has yet to be reported.

More recently, NTRK1 point mutations were observed in three patients with acute erythroid leukemia from a cohort of 159 patients that were sequenced [79]. These mutations—H498R, G617D, and H766R—were located in the kinase domain of the receptor. To assess the leukemogenic potential of these NTRK1 mutations, wildtype and mutated NTRK1 were expressed in lineage-negative hematopoietic stem and progenitor cells from wildtype or TP53 mutated mice. The coexpression of mutated NTRK1^H498R/ G617D/ H766R^ with TP53^R172H^ culminated in an extremely penetrant form of erythroid leukemia [79]. However, a single NTRK1 mutation in the absence of mutated TP53 had no effect on the overall survival of these animals. Likewise, a single TP53 mutation resulted in a mild form of disease, implicating that the cooccurrence of TP53 and NTRK1 mutations explains the underlying oncogenicity.

### Changes in mRNA and protein expression

In 1996, Kaebisch et al. reported the association of Trk expression and leukemia [80]. After evaluating gene expression in 59 patients with acute myeloid leukemia (AML), they observed upregulation of NTRK1 transcripts in 44% of the patients in the cohort. Following this initial study, a series of studies reported a role of Trk receptors and their respective ligands in various stages of hematopoiesis [81, 82]. Trk receptors promote proliferation and survival of erythroblasts, dendritic cells, lymphocytes, monocytes, and macrophages [83]. Specifically, NTRK2 is highly expressed in immature thymocytes and its expression progressively declines throughout the T cell maturation process [84]. Despite these studies, the functional role of Trk receptors in hematopoiesis is not completely understood.

Since then, many studies have reported overexpression of NTRK receptors in a variety of hematological malignancies. Overexpression of NTRK1 was observed in AML patient samples harboring the AML1–ETO fusion protein, generated by t(8;21) [85]. While it is uncertain what provides these AML1–ETO-expressing leukemia cells a growth advantage, one hypothesis is their crosstalk with bone marrow stromal cells that express nerve growth factor (NGF). NGF binds to its cognate receptor, TrkA, and may therefore drive leukemia.

In a prospective study of 94 adult patients with de novo or secondary AML, ALL, or acute undifferentiated leukemia, expression of at least one NTRK receptor was seen in 55% of the analyzed cases [78]. While coexpression of two or more NTRK receptors was observed on AML blasts, ALL blasts exclusively expressed NTRK2.

After mining large publicly available datasets (e.g., Microarray Innovations in Leukemia (MILE) study), Herbrich et al. (2018) reported that expression of NTRK1 was significantly higher in a combined group of 456 patients with AML when compared with normal CD34^+^ bone marrow cells [86]. Separating AML samples by cytogenetic subtypes, they saw the highest expression of NTRK1 mRNA in patients with t(8;21) and inv(16)/t (16;16). The increase in NTRK1 mRNA that they observed in patients with t(8;21) was in line with Mulloy et al., who observed a similar trend after analyzing 262 primary AML patient samples [85].

Recent work from our laboratory has uncovered a critical dependency on Trk signaling in TP53^mutant^ venetoclax-resistant AML cells [87]. To identify essential target genes and pathways contributing to venetoclax resistance in AML, we performed a genome-wide CRISPR/Cas9 screen on a patient-derived AML cell line. We identified TP53, BAX, and PMAIP1 as key genes whose inactivation conferred resistance to venetoclax. Moreover, TP53 knockout cells were found to gain sensitivity to a panel of Trk inhibitors, suggesting a dependency on Trk-mediated activation for the survival of TP53 mutant cells. We saw a similar correlation in TP53-mutant AML patient samples. Our findings were in line with the work of Iacobucci et al. who recently demonstrated that the cooccurrence of NTRK1 and TP53 mutations promoted the development of an aggressive erythroid leukemia [79]. Our collective findings highlight a new potential clinical utility of Trk inhibitors in leukemia harboring TP53 mutations.

Upregulation of the Trk receptors has also been reported in mastocytosis, a subcategory of myeloid neoplasms. Peng et al. (2013) were the first to show elevated expression of TrkB and TrkC receptors on the mast cells of patients with mastocytosis [88]. Thereafter, Yang et al. (2014) demonstrated that activation of TrkB by BDNF in murine hematopoietic stem and progenitor cells induces a disease phenotype that mimics the clinical presentation of mastocytosis [89]. More recently, they showed that activation of the TrkA receptor triggers the onset of mastocytosis in mice and confers drug resistance [90].

While NTRK receptors can be activated via multiple mechanisms, proliferation, and survival of cells harboring NTRK alterations is dependent on similar downstream signaling cascades as those activated by BCR–ABL1 in CML (i.e., MAPK, PI3K/AKT, and JAK/STAT) [17, 24, 29, 86, 91–93]. Such patterns of signaling contribute to the observed phenotype.

## NTRK and EN fusion expressing cell line models

Despite the identification of NTRK mutations and fusions in leukemic patients, there are a limited number of cell culture models available to study the role of Trk receptors in leukemogenesis and validate potential inhibitors. Based on mRNA and protein expression analysis, Kaebisch et al. (1996) reported that the following myeloid leukemia cell lines—HEL, K562, and KG-1—expressed NTRK3 [80]. Moreover, they found that treating the human promyelocytic cell line, HL-60, with tetradecanoylphorbol 13-actetate induced expression of NTRK3 in these cells. After performing a comprehensive and systematic review of TrkA signaling in leukemia, another study has identified 11 cell lines that have detectable levels of NTRK1 transcripts [86]. The highest expression was observed in megakaryoblastic (i.e., CMK), erythroleukemic (i.e., TF-1), and CML (i.e., K562) cell lines [86].

Three main AML cell lines have been cited in the literature to study EN fusions: IMS-M2, M0-91, and AP-1060 [52, 94, 95]. Bone marrow cells taken from an EN-positive AML-M2 patient gave rise to the IMS-M2 cell line [52]. After screening over 40 AML cell lines with mass spectrometry and sequencing, Gu et al. (2007) identified M0-91 as an EN-expressing cell line with increased phosphorylation of TrkB and TrkC. siRNA-mediated knockdown of the EN fusion in these cells decreased their growth and viability, suggesting that the EN fusion was essential for the growth and survival of M0-91 cells [94]. Treatment of M0-91 cells with an IGF1R inhibitor also promotes degradation of the endogenous fusion protein [96]. These results suggest that the M0-91 EN fusion possesses signaling and regulatory properties similar to those observed in other engineered cell line models. Using genomic and transcriptomic microarray-based profiling, Chen et al. (2018) have recently shown the presence of the EN fusion in an acute promyelocytic leukemia (APML) cell line, called AP-1060 [95].

## NTRK inhibition in hematological malignancies

The discovery of imatinib for the treatment of CML has shifted the paradigm of cancer treatment toward precision oncology [5, 97, 98]. It was the first small-molecule protein-kinase inhibitor that was designed to target a specific kinase fusion, BCR–ABL1. Its success has paved the way for development of other kinase-specific inhibitors and curtailed the use of empiric chemotherapy for CML patients. Among these inhibitors, Trk inhibitors have attracted considerable attention over the past few years owing to their remarkable efficacy in patients harboring NTRK fusions in early clinical trials [12, 27]. As mentioned earlier, the presence of the EN fusion is pathognomonic for several rare solid tumor malignancies [24, 26, 29–31], further underscoring the need for Trk inhibitors. Despite the rarity of NTRK-mediated clinical cases reported in leukemias, the positive clinical evidence observed in solid tumors provides an impetus to investigate the efficacy of such inhibitors in these tumors. The available preclinical and clinical studies that have investigated the potency of these inhibitors in hematologic malignancies are summarized below (Table 1).

In 2009, Li et al. evaluated apoptosis in cultured leukemic cells obtained from four patients with AML [78]. These cells were exposed for ~18h to varying concentrations (100–400 nM) of K252a, a previously validated nonselective Trk inhibitor (indolocarbazole analogue) [99], resulting in a 65% reduction of viable cells and dephosphorylation of NTRK receptors. Using an AML patient-derived mouse xenograft, they also saw enhanced survival of mice following treatment with AG879, a TrkA inhibitor [78].

Minimal response to standard chemotherapy seen in two AML patients [53, 58] harboring the EN fusion prompted Chi et al. (2012) to consider evaluating midostaurin, a broad spectrum kinase inhibitor [100–102]. Treatment with 100 nM of midostaurin inhibited EN activity in AML cell lines (i.e., IMS-M2, M0-91) carrying the fusion and Ba/F3 cells stably expressing the fusion. Following 8 h of treatment, phosphorylation of STAT5, AKT, and MAPK was suppressed and apoptosis was induced. This was the first study to show that small-molecule kinase inhibitors could serve as a promising avenue to treat NTRK-driven leukemias.

Although initially approved against ALK fusions in patients with non-small cell lung cancer (NSCLC) [103], crizotinib, another small-molecule kinase inhibitor was evaluated on NTRK-mediated cancers with the first case being a patient with NSCLC harboring the MPRIP–NTRK1 fusion [22]. When tested on IMS-M2 and M0-91 AML cell lines, crizotinib blocked proliferation of EN-dependent tumor cells, decreased phosphorylation of downstream signaling, and impacted growth of tumor xenografts [104]. Three additional inhibitors (imatinib, ponatinib, and NVP-TAE684) were also considered but only crizotinib demonstrated nanomolar potency in the cell-based assay.

Similarly, the EN fusion of a patient with Ph-like ALL was sensitive to crizotinib [60]. This study also assessed the efficacy of crizotinib in a xenograft model in which EN^+^ cells from the Ph-like ALL patient were engrafted into immunodeficient mice. Ex vivo cytotoxicity assay data from these mice showed sensitivity to crizotinib whereas imatinib had no effect.

In another case, a 37-year-old relapsed-AML patient with an abundance of EN transcripts was sensitive to BMS-754807, an IGF-IR/TrkC inhibitor [59]. This inhibitor was also effective in blocking EN-mediated transformation in cell lines models [105] as well as inhibiting viability of M0-91 cells [96]. Interestingly, an EN fusion found within a cell culture model of APML, [95] a subtype of AML, was hypersensitive to AZ-23, a selective NTRK inhibitor that has been previously validated in a Trk-expressing xenograft model of neuroblastoma [106].

Results from basket trials performed on entrectinib (RXDX-101), a selective pan-Trk, ROS1, and ALK inhibitor have garnered much attention. Entrectinib demonstrated robust antitumor activity across a broad range of solid tumors regardless of histology [27,107]. It was well tolerated, with mainly Grade 1 and 2 adverse events that were reversible with dose modification. Given entrectinib’s therapeutic profile, Yang et al. (2017) showed that treatment with entrectinib decreased activation of TrkA in mast cell lines (HMC-1, HMC-1.2), primary mast cells from patients with systemic mastocytosis, and in mice xenotransplanted with HMC-1 cells. Moreover, they showed that inhibition of Trk signaling via entrectinib restored sensitivity to KIT inhibition in their in vitro and in vivo studies [90].

Another study reported that entrectinib inhibited cell proliferation, at subnanomolar concentrations, of IMS-M2 and M0-91 cells (0.47 and 0.65 nM, respectively) and induced apoptosis [108]. Entrectinib was 6–158-fold more potent than other tested Trk inhibitors (i.e., crizotinib, larotrectinib, and belizatinib (TSR-011)). Phosphorylation of the EN fusion and downstream signaling mediators was inhibited by entrectinib in a dose-dependent manner. Lastly, in animal models (mice and zebrafish), entrectinib treatment resulted in tumor regression. In August 2019, entrectinib received FDA approval for adults and pediatric patients with solid tumors harboring NTRK mutations [15].

Likewise in a cohort of 17 tumor types, larotrectinib, the most selective pan-Trk inhibitor, blocked Trk signaling with an overall response rate of 80% in patients. Based on these data, in November 2018 larotrectinib received Breakthrough Therapy FDA approval for adult and pediatric patients with solid tumors bearing NTRK fusions [12]. Given the efficacy of larotrectinib in solid tumors, its ability to inhibit the EN fusion in a murine xenograft model of Ph-like B-ALL and Ba/F3 cell assay was evaluated [61]. In all cases, treatment with larotrectinib reduced the tumor burden, splenic weight, and decreased phosphorylation of ERK1/2, STAT3, and STAT5 with robust response seen following 24 h. Similar trends were seen with PLX7486, a NTRK, CSF1R, and AURK inhibitor. However, larotrectinib was much more efficacious. In comparison to crizotinib, within their Ba/F3-EN model, larotrectinib was ~12 times more potent (17 nM vs. 205 nM, respectively) [61].

Taylor et al. (2018) also evaluated larotrectinib’s activity on Trk fusions found in patients with AML, histiocytosis, and multiple myeloma in a cell-based proliferation assay and on an AML patient-derived xenograft containing ETV6–NTRK2 cells. In all cases, larotrectinib reduced expression and Trk activity [66]. Based upon the xenograft model results, larotrectinib was given twice daily to a 77-year-old man with refractory secondary AML possessing an ETV6–NTRK2 fusion. The patient achieved a partial remission with changes in ETV6–NTRK2 fusion abundance mirroring clinical response [66]. Although these results are from a single case report, they suggest a potential clinical utility of Trk inhibition in hematological malignancies harboring Trk fusions and highlight a previously unrecognized role of the ETV6–NTRK2 fusion. Due to the inherent differences in biology between solid and liquid tumors, a tailored basket trial or other unique clinical trial designs may be required specifically for Trk fusion positive leukemias and lymphomas.

Our recent work in a venetoclax-resistant cell line model system uncovered a role for NTRK in TP53 null cells [87]. TP53 knockout cell lines were found to be highly sensitive to a number of Trk inhibitors such as entrectinib, larotrectinib, GW-2580, crizotinib, AZD 1480, and foretinib. These results, along with confirmatory western blots in cell lines and primary patient samples identified upregulation of Trk protein in the context of TP53 loss of function and a concomitant gain of sensitivity to Trk inhibitors. Similar to our work, mice harboring NTRK1 and TP53 comutations developed a highly aggressive erythroid leukemia that was very responsive to larotrectinib, further highlighting the relevance of Trk inhibitors for clinical trials in patients with hematological malignancies [79].

Apart from the aforementioned inhibitors, many others have been validated in solid tumors bearing NTRK fusions or mutations. These include cabozantinib [109], foretinib [109], merestinib [110], and nintedanib [109] among others. Moreover, IGF1R/IR pathway inhibitors, in particular, have shown to be effective at blocking EN activity by initiating a process that results in the ubiquitylation and proteasomal degradation of the fusion protein itself [96].

Amino acid substitutions involving the solvent front, activation loop DFG motif, or gatekeeper residues have conferred resistance to a variety of tyrosine kinase inhibitors [111–113]. In CML, the classic gatekeeper mutation, BCR–ABL^T315I^ interferes with imatinib’s ability to bind the ABL kinase, rendering it ineffective [91, 112]. Similar mechanisms of resistance have been recently reported for entrectinib and larotrectinib in the setting of colorectal [114] and MASC [26] tumors. As such, second generation NTRK inhibitors that overcome this acquired resistance are currently in development. These inhibitors include LOXO-195 [115] and repotrectinib (TPX-0005) [116]. Advancements in molecular profiling and the occurrence of NTRK mutations in liquid tumors, calls for the validation of these inhibitors in hematologic malignancies.

## Conclusion

The success of imatinib not only improved the prognosis for patients with CML but ushered an era of precision oncology—where knowledge of the underlying biology is key to efficient drug development. Though Trk oncofusions were among the first oncogenes to be identified following BCR–ABL1, they often went unnoticed due to their low prevalence among various solid cancers. However, this is no longer the case. Recent advances in deep sequencing technologies and immunohistochemical techniques provide promising avenues to identify Trk alterations [12, 27, 117, 118]. In many ways, such methods have negated the notion that alterations in Trk receptors are uncommon. Mechanistic studies have further shown the importance of Trk signaling in driving leukemia. The success of early clinical trials with larotrectinib and entrectinib as well as the different studies discussed above suggest that oncogenic Trk aberrations are amenable to targeted inhibition and hold the promise of providing clinical benefit in a variety of hematologic malignancies. While it is difficult to predict the efficacy of Trk inhibition in patients, our work, and that of others provide a rationale for the need to initiate trials focused on Trk inhibition in liquid tumors.

## Figures and Tables

**Table 1:** Summary of inhibitors targeting NTRK in hematological malignancies

Drug name	Malignancy	PMID	Publication year
AG-879	AML	19059881	2009
AZ-23	APML	29119387	2018
AZD-1480	AML	31048320	2019
Belizatinib (TSR-011)	AML	29237803	2017
BMS-754807	AML	24056683	2013
		29903916	2018
Crizotinib	AML	23811600	2013
	Ph-like B-ALL	25207766	2014
	AML	29237803	2017
	Ph-like B-ALL	29880614	2018
	AML	31048320	2019
Entrectinib	Mastocytosis	29088753	2017
	AML	29237803	2017
	AML	31048320	2019
Foretinib	AML	31048320	2019
GW-2580	AML	31048320	2019
K252a	AML	19059881	2009
Larotrectinib	Ph-like B-ALL	29880614	2018
	AML	29920189	2018
	AML	29920189	2018
	ECD	29920189	2018
	Multiple Myeloma	29920189	2018
	Multiple Myeloma	29920189	2018
	AEL	30926971	2019
	AML	31048320	2019
Midostaurin (PKC412)	AML	23131561	2012
PLX7486	Ph-like B-ALL	29880614	2018

This table provides a summary of inhibitors targeting NTRK receptors in hematological malignancies that have been investigated in preclinical and clinical studies
